# Chromosome-level genome assembly and annotation of the prickly nightshade *Solanum rostratum* Dunal

**DOI:** 10.1038/s41597-023-02247-3

**Published:** 2023-06-01

**Authors:** Yue Zhang, Wenchao Guo, Zhili Yuan, Zhen Song, Zhonghui Wang, Jinhui Gao, Weidong Fu, Guoliang Zhang

**Affiliations:** 1 Institute of Environment and Sustainable Development in Agriculture, Chinese Academy of Agricultural Sciences, Beijing, 100081 China; 2 Key Laboratory of Intergraded Management of Harmful Crop Vermin of China Northwestern Oasis, Ministry of Agriculture and Rural Affairs/Institute of Plant Protection, Xinjiang Academy of Agricultural Sciences, Urumqi, 830091 China

**Keywords:** DNA sequencing, Sequence annotation, Comparative genomics, Genome duplication, Genome evolution

## Abstract

The prickly nightshade *Solanum rostratum*, an annual malignant weed, is native to North America and has globally invaded 34 countries, causing serious threats to ecosystems, agriculture, animal husbandry, and human health. In this study, we constructed a chromosome-level genome assembly and annotation of *S. rostratum*. The contig-level genome was initially assembled in 898.42 Mb with a contig N50 of 62.00 Mb from PacBio high-fidelity reads. With Hi-C sequencing data scaffolding, 96.80% of the initially assembled sequences were anchored and orientated onto 12 pseudo-chromosomes, generating a genome of 869.69 Mb with a contig N50 of 72.15 Mb. We identified 649.92 Mb (72.26%) of repetitive sequences and 3,588 non-coding RNAs in the genome. A total of 29,694 protein-coding genes were predicted, with 28,154 (94.81%) functionally annotated genes. We found 99.5% and 91.3% complete embryophyta_odb10 genes in the pseudo-chromosomes genome and predicted gene datasets by BUSCO assessment. The present genomic resource provides essential information for subsequent research on the mechanisms of environmental adaptation of *S. rostratum* and host shift in Colorado potato beetles.

## Background & Summary

**Fig. 1:**
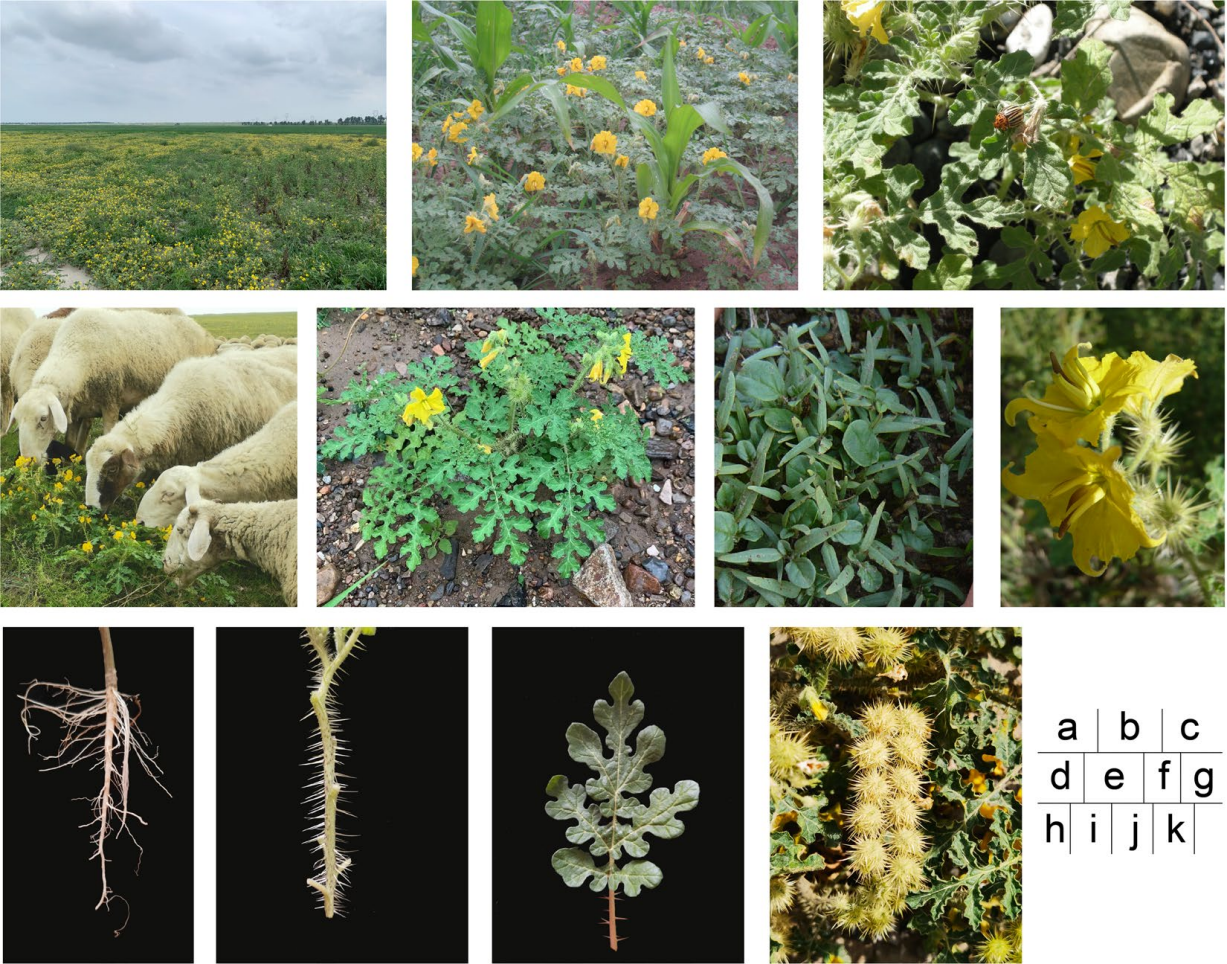
Morphological characteristics of *Solanum rostratum* (**a**) habits in the grassland, (**b**) habits in the corn field, (**c**) infested by Colorado potato beetle in the field, (**d**) damage to livestock, (**e**) whole plant, (**f**) seedling, (**g**) flower, (**h**) root, (**i**) stem, (**j**) leaf, and (**k**) fruit.

The prickly nightshade, *Solanum rostratum* Dunal (Solanales: Solanaceae), an annual plant, is an invasive alien malignant weed which classified as an “agricultural weed”, an “environmental weed”, and a “noxious weed” in the Global Compendium of Weeds^1^. In China, it is listed as an entry quarantine pest and key management alien invasive species. This species has a fast growth rate and strong reproductive ability, whose seed production reaching 78,500 seeds per plant^2^. High competitiveness in light, water, nutrients, ecological niche, and other resources results in reduced agricultural land production, loss of native species’ competitive advantage, and decrease in biodiversity. In addition, the densely covered narrow and long prickles on the surface of the stem, leaf, calyx, and fruit can be mixed with fodder to hurt the oral cavity and gastrointestinal digestive tract of livestock. Moreover, the neurotoxin solanine present in whole plants can cause livestock poisoning^3^. It is also the host of the Colorado potato beetle *Leptinotarsa decemlineata*^4^, which is the most destructive pest on potatoes, the tomato golden mottle virus^5^, and the tomato severe leaf curl virus^6^. Thus, the invasion of *S. rostratum* seriously threatens the local ecological environment, agricultural production, grassland animal husbandry, biodiversity, and human health (Fig. 1).

The extremely strong ecological adaptability (could survive in the wasteland, grasslands, overgrazing pastures, roadside, garbage dumps, orchards, courtyards, irrigation ditches, and river beaches)^7^ and stress resistance (barren, drought, wet, and salt^8^) facilitate *S. rostratum* to spread and establish in a new environment as a dominant species. Native to North America^9^, *S. rostratum* is widely distributed in 34 countries and regions, including North America, Asia, Africa, Europe, and Oceania^10^. In China, since its first detection in 1981 in Chaoyang City, Liaoning Province^11^, it has spread to nine provinces and 54 counties within 30 years through water flow, wind, livestock trade, sand transportation, and other vectors.

Alien invasive plants usually can adapt to the new ecological environment and establish and expand populations within a short period^12^, which will seriously negatively impact the local ecosystem. High-quality reference genomes could help us profoundly comprehend and screen the genetic basis and variations associated with important traits and adaptation under different ecological and environmental conditions. Technological advances, including long-read sequencing by Pacific Biosciences (PacBio) or Oxford Nanopore Technologies (ONT), the chromosome conformation capture technique (Hi-C), and BioNano optical maps, have facilitated genome sequencing, assembly, and annotation, leading to the rapid expansion of the quantity and quality of public plant genomes in the past 20 years^13,14^. For the nightshade family, Solanaceae, which comprises approximately 90 genera and 3,000–4,000 species^15^, a total of 7 genera, 46 species, and 170 genomes have been reported. However, all previous genomic studies have focused on horticultural crops and their related wild species (for example, the cultivated tomato *Solanum lycopersicum*^16^ and the wild relative *Solanum pimpinellifolium*^17^, potato *Solanum tuberosum*^18^, hot pepper *Capsicum annuum*^19^, and eggplant *Solanum melongena*^20^), model plant organisms (tobacco *Nicotiana tabacum*^21^), ornamental flowers (*Petunia inflata* and *Petunia axillaris*^22^), and herbs (*Datura stramonium*^23^ and *Lycium barbarum*^24^). So far, the genome of the solanaceous malignant weed remains unsequenced. Therefore, a chromosome-level reference genome of *S. rostratum* is an essential resource to further elucidate the pathway and genes involved in ecological environment adaptation under different stresses, solanine biosynthesis, host shift from native host prickly nightshade to potato for Colorado potato beetle, etc., by integrating comparative genomics, functional genomics, metagenomics, and population genomics.

**Fig. 2:**
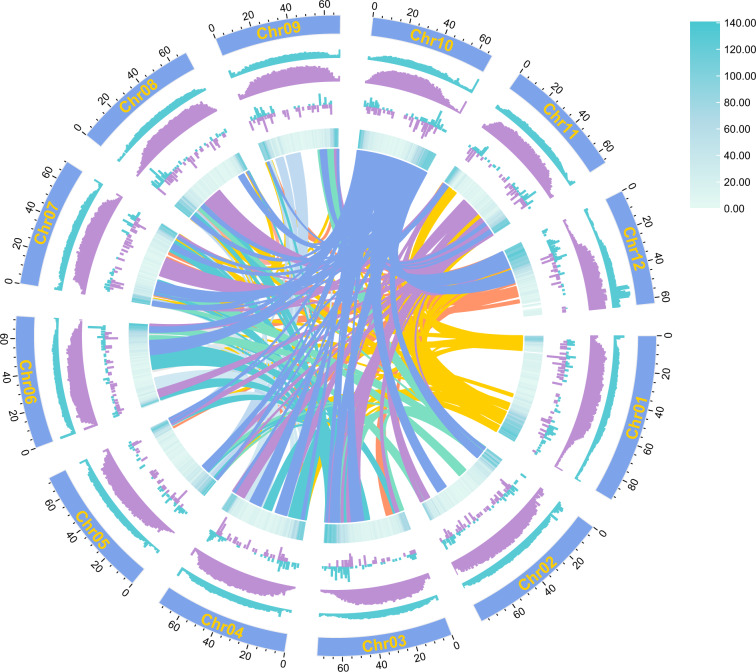
Chromosome-scale assembly genomic landscape of *Solanum rostratum*. Circos plot from the outer to the inner layers represents the following: (1) 12 pseudo-chromosomes length at the Mb scale; (2) GC content per Mb; (3) repeat density per Mb; (4) Copia (blue) and Gypsy (purple) LTR retroelement density per Mb; (5) gene density per Mb; and (6) center: intra-genomic syntenic blocks of *S. rostratum*.

In this study, we constructed and annotated a high-quality chromosome-level reference genome using integrated sequencing data (Fig. 2). We performed an initial *de novo* assembly into a contig-level genome by Hifasm^25^ using PacBio High fidelity (HiFi) long-reads. Valid Illumina Hi-C paired-end reads were used to generate chromosome-level assemblies using the HiC-Pro pipeline^26^. After masked repeat sequences, three strategies were integrated to annotate the gene structure by EVidenceModeler (EVM)^27^, including homologous prediction against closely related species, transcriptome-based prediction using the transcripts generated from PacBio Isoform-Sequencing (Iso-seq) long-reads and Illumina Paired-end RNA-seq short-reads by Program to Assemble Spliced Alignments (PASA) pipeline^28^, and *ab initio* prediction based on the characteristics of genomic sequence data. After annotating protein-coding gene functional and protein domains against a related database, the completeness and quality of the genome assembly and annotation were evaluated by Benchmarking Universal Single-Copy Orthologs (BUSCO)^29^ analysis and genome mapping and coverage rates using Illumina Paired-end short-reads. These results indicate that the present genome assemblies and annotations are contiguous and accurate. Furthermore, comparative genomic analysis was conducted with other nineteen solanaceous species to provide insight into their phylogenetic relationship, divergence time, whole-genome duplication (WGD) events along the solanaceous speciation, and genomic evolutionary history. Thus, the present *S. rostratum* genomic resource will be a foundation for subsequent research on this weed.

## Methods

### Plant material collection and preparation

Healthy mature plants of *S. rostratum* were collected from the wasteland in Chaoyang City, Liaoning Province, China (120.504360° E, 41.604752° N) in August 2021. After washing with deionized water, the roots, stems, leaves, flowers, and fruits were harvested. All the tissues were put into liquid nitrogen immediately and preserved in an ultra-low temperature freezer until use.

### DNA library construction and genome sequencing

High molecular weight genomic DNA was extracted from tender leaves with a modified 2 × cetyltrimethylammonium bromide (CTAB) method^30^. Approximately 200 mg of tender leaves were ground to powder using liquid nitrogen and then added to 800 μL of CTAB lysis buffer in a 2.0-mL tube. After incubation at 65 °C for 60 min, 800 μL of phenol/ chloroform/ isopentanol (25:24:1) was added and centrifuged at 12,000 rpm for 10 min. The supernatant was extracted into another 2.0-mL tube with an equal volume of chloroform/isopentanol (24:1). After mixing by gentle inversion, the tube was centrifuged at 12,000 rpm for 10 min. The supernatant was extracted to another tube with 0.6 times the volume of precooled (−20 °C) isopropanol. After being placed at −20 °C for over 2 h, the tube was centrifuged at 12,000 rpm for 10 min. The pellet was washed twice with 75% ethanol and dissolved in 50 µL of DNase and RNase-free Water for further study.

For the Illumina whole-genome shotgun raw sequencing, the genomic DNA was randomly fragmented, and a library with an average insert size of 350 bp was constructed using the Illumina TruSeq Nano DNA Library Prep Kit (Illumina, USA) following the manufacturer’s instructions. The library was sequenced on the Novaseq 6000 Platform set in the PE150 program, generating a total of 103.47 Gb of raw data. After filtering by fastp v0.12.4^31^ with default to remove low quality and short reads and cut adapters and polyG, 102.14 Gb (113.69×) clean data were retained for the genome size estimation (Table 1).

**Table 1:** Statistics of sequencing data for *Solanum rostratum* genome assembly and annotation.

Platform	Type	sample	Molecule	Total clean data	Coverage	Usage	SRA accession number
Illumina NovaSeq	PE	Leaf	DNA	102.14 Gb	113.69×	correction	SRR23354532
PacBio HiFi	CCS	Leaf	DNA	25.83 Gb	28.75×	*de novo* assembly	SRR23354533
Illumina Hi-C	PE	Leaf	DNA	102.09 Gb	113.63×	chromosome-level assembly	SRR23354531
PacBio Iso-seq	Subreads	Mixed	RNA	19.81 Gb	22.05×	gene structure annotation	SRR23354525
Illumina NovaSeq	PE	Root	RNA	6.00 Gb	6.90×	gene structure annotation	SRR23354529
Illumina NovaSeq	PE	Stem	RNA	7.14 Gb	8.20×	gene structure annotation	SRR23354528
Illumina NovaSeq	PE	Leaf	RNA	6.93 Gb	7.97×	gene structure annotation	SRR23354530
Illumina NovaSeq	PE	Flower	RNA	6.92 Gb	7.96×	gene structure annotation	SRR23354527
Illumina NovaSeq	PE	Fruit	RNA	6.94 Gb	7.98×	gene structure annotation	SRR23354526

The PacBio Sequel II System, based on single-molecule real-time (SMRT) sequencing technology under the Circular Consensus Sequencing (CCS) model, was used for whole-genome sequencing. The DNA template was sheared by g-TUBE (Covaries, USA) to an average size of 15–20 kb, and the target DNA fragments were obtained using BluePippin^TM^ Size-Selection System (Sage Science, USA). The library was constructed using SMRTbell Template Prep Kit 1.0 (Pacific Biosciences, USA) following the procedure and loaded onto PacBio Sequel™ Systems to read the sequence. Finally, approximately 366.02 Gb subreads were obtained with an average length of 13.59 kb and an N50 length of 15.25 kb after removing adaptors in polymerase reads (Table 1).

### RNA library construction and transcriptome sequencing

Total RNA was isolated from the roots, stems, leaves, flowers, and fruits, respectively, using the standard TRIzol protocol (Invitrogen, USA)^32^. Approximately 100 mg of tissue was ground to powder using liquid nitrogen, and then 1000 μL of TRIzol was added in a 2.0-mL tube. After allowing the solution to stand for approximately 5 min, 200 μL of chloroform was added, shaken vigorously for 30 s, and allowed to stand for 3 min. After centrifugation at 12,000 rpm for 15 min at 4 °C, the upper aqueous phase was extracted to another 1.5-mL tube with 500 μL of isopropanol and then mixed by gently inverting. After standing for approximately 10 min, the tube was centrifuged at 12,000 rpm for 10 min. The supernatant was removed, and the pellet was washed twice with 75% ethanol and dissolved in 50 µL of DNase and RNase-free Water for further study.

For the Illumina paired-end reads sequencing, the mRNA was synthesized to cDNA, and five libraries were constructed with an insertion size of 350 bp using a TruSeq RNA library preparation kit (Illumina, USA) following the manufacturer’s instructions. Whole-genome shotgun raw sequencing was performed using the Novaseq 6000 Platform set in the PE150 program. In total, 32.91 Gb of clean data were generated from the RNA-seq library after filtering using fastp^31^ (Table 1).

For Iso-seq under the CCS model, the RNA samples extracted from root, stem, leaves, flowers, and fruits were equally mixed for sequencing. cDNA was synthesized using a Clontech SMARTer PCR cDNA Synthesis Kit (Takara Biotechnology, China). Then, the SMRTbell library (cDNAs length over 4 kb) was constructed using the Pacific Biosciences SMRTbell template prep kit (Pacific Biosciences, USA) and sequenced on the Pacific Bioscience Sequel II platform. A total of 19.81 Gb subreads were obtained with an average length of 2,562 bp and an N50 length of 3,005 bp after removing adaptors in polymerase reads (Table 1). The exported subreads were analyzed using packages of SMRT link v10.1, including highly accurate consensus sequence calling using package ccs v6.0.0 (https://github.com/PacificBiosciences/ccs), primer removal and demultiplexing using package lima v2.1.0 (https://github.com/pacificbiosciences/barcoding/), polyA tail and artificial concatemers removal using package isoseq3 v3.4.0 (https://github.com/PacificBiosciences/IsoSeq), and clustering and polishing using package isoseq3 v3.4.0. Finally, approximately 387.83 Mb high-quality consensus isoform sequences were generated with an average length of 3,843 bp.

### Contig-level genome assembly

The in-built High-Quality Region Finder (HQRF) was used to identify the longest high-quality regain for each read of exported subreads according to the signal noise ratio (SNR). HiFi reads were then generated from filtered subreads using the CCS model of SMRT link v10.1 with the following parameters: --maxLength = 50000, --minPasses = 3, and --minPredictedAccuracy = 0.99. The sequences in fastq.gz were converted from the BAM file using bam2fastx v1.3.1 (https://github.com/pacificbiosciences/bam2fastx/). 25.83 Gb (28.75×) of CCS reads were obtained with an average length of 15.34 kb and an N50 length of 15.78 kb (Table 1). Then, Hifiasm v0.16.0^25^ was used to assemble the genome into contigs with default parameters. To check for the potential contaminant sequences, assembled contigs were classified using Kraken2 against the custom database^33^. Four contigs were identified as bacteria (904,041 bp, 0.10%), which were flagged and removed from the final assembly. After removal, the final contig-level assembly was submitted to the NCBI independent contamination check to confirm the result, resulting in an 898.42 Mb contig-level genome consisting of 113 contigs and an N50 length of 62.00 Mb (Table 2).

**Table 2:** Statistics of the *Solanum rostratum* genome assembly.

Feature	Metric
**Hifiasm-derived contigs**	
Number of contigs	113
Total length of contigs	898,418,632 bp
Longest contig	92,793,329 bp
contig N50	62,003,212 bp (contig number = 7)
Contig N60	51,582,526 bp (contig number = 8)
Contig N70	44,575,705 bp (contig number = 10)
Contig N80	31,624,497 bp (contig number = 13)
Contig N90	12,481,291 bp (contig number = 17)
**Hi-C scaffolded assembly**	
Number of scaffolds	224
Total length of scaffolds	869,692,437 bp
Longest scaffold	92,283,834 bp
Scaffold N50	72,149,870 bp (scaffold number = 6)
Scaffold N60	69,863,894 bp (scaffold number = 8)
Scaffold N70	68,611,519 bp (scaffold number = 9)
Scaffold N80	67,684,033 bp (scaffold number = 10)
Scaffold N90	63,146,500 bp (scaffold number = 12)
GC content	36.99%

### Hi-C library construction and pseudo-chromosome anchoring

**Fig. 3:**
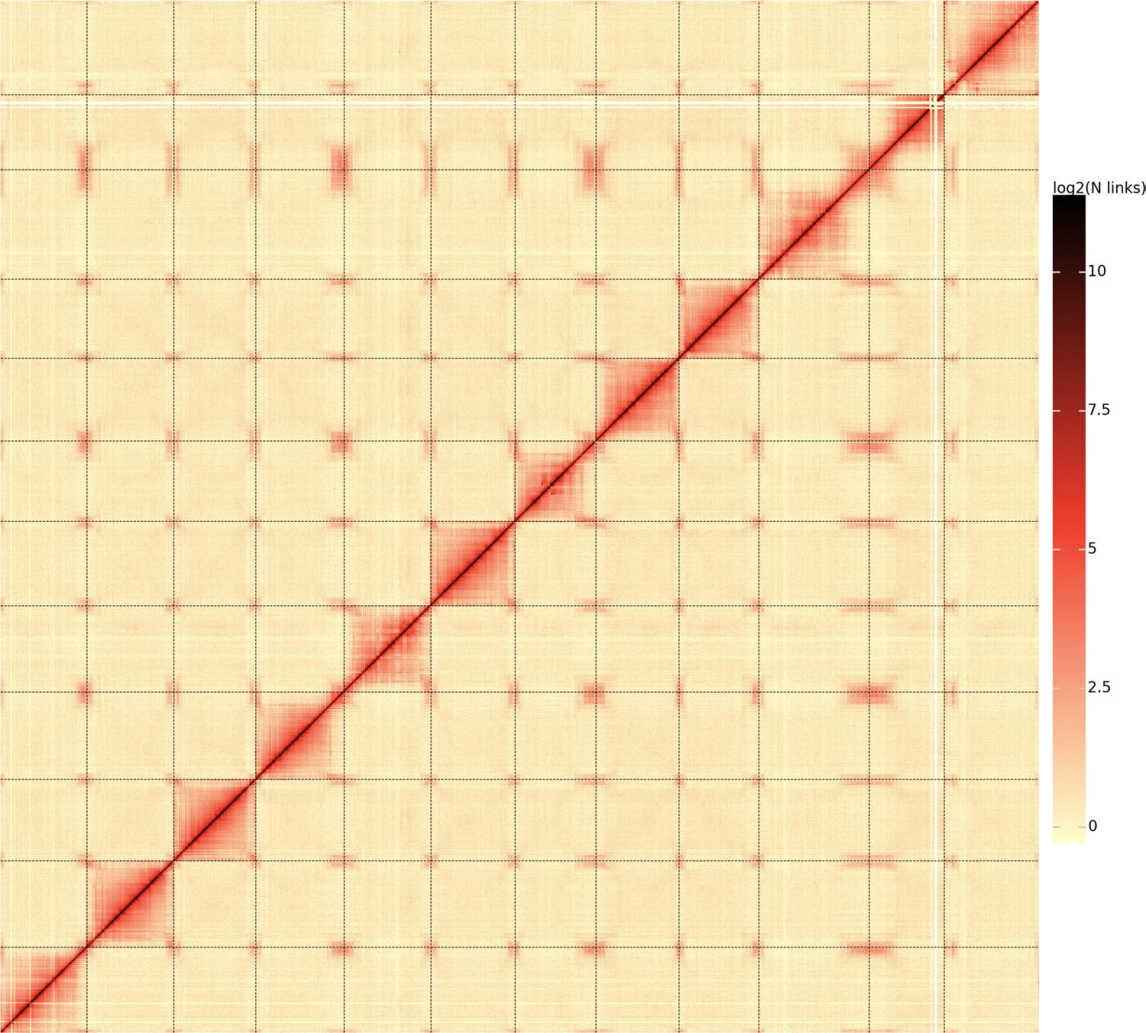
Heat map of genome-wide Hi-C intra-chromosome interactions in *Solanum rostratum*. The interaction density is measured by the number of supporting Hi-C reads and illustrated by the color bar from dark red (high density) to light pink (low density).

Tender leaves were cut into approximately 2-cm^2^ pieces for cellular protein cross-linking in 2% formaldehyde. The isolated DNA was purified, digested with *Dpnii* restriction enzyme, tagged with biotin-14-dCTP, sheared into 300–600 bp fragments, and blunt-end-repaired. Then, the Hi-C library was sequenced using the Illumina NovaSeq platform, which generated 100.16 Gb filtered clean data (113.63×) to anchor contigs into pseudo-chromosomes (Table 1). The cleaned Hi-C sequencing data were aligned on the contig assembly using bowtie2 v2.2.5^34^ to obtain the unique mapped paired-end reads using the following parameters:--very-sensitive -L 20--score-min L, -0.6, --0.2 --end-to-end --reorder --rg-id BMG --phred33-quals -p 5. Quality control of read alignment and pairing was conducted using HiC-Pro v2.7.8^26^ to discard low-quality alignment, singleton, multiple hits, and invalid pairs. A total of 156,223,644 valid paired-end reads were used to build the interaction matrices and scale up the primary genome assembly in contigs to chromosome-scale scaffolds (pseudo-chromosomes). A total of 869.69 Mb of the contig-level assembled sequences (96.80% anchored rate) were anchored and orientated onto 12 pseudo-chromosomes, which was consistent with the karyotype (2n = 24) analysis^35^, with lengths ranging from 63.15 to 92.28 Mb (Table 3). In summary, the size of the pseudochromosome-level *S. rostratum* genome that was obtained was 869.69 Mb with 212 unanchored contigs (total length 28.73 Mb), with a contig N50 of 72.15 Mb (Table 2). To validate the correction of the pseudo-chromosome anchoring result, the pseudo-chromosomes were divided into bins of equal size in 50 kb to construct genome-wide interaction matrices based on the interaction signals between each pair of bins. The interaction matrix heatmap was visualized using HiCPlotter v0.6.6^36^ (Fig. 3).

**Table 3:** Statistics of *Solanum rostratum* genome assembly result by Hi-C.

Chromosome	Total length	ATCG base number	N base number	Gap ratio
Chr01	92,283,834	92,280,834	3,000	0.003251%
Chr02	79,062,878	79,056,378	6,500	0.008221%
Chr03	73,759,959	73,758,959	1,000	0.001356%
Chr04	73,025,073	73,024,073	1,000	0.001369%
Chr05	72,660,254	72,653,754	6,500	0.008946%
Chr06	72,149,870	72,137,370	12,500	0.017325%
Chr07	70,619,802	70,617,802	2,000	0.002832%
Chr08	69,863,894	69,863,894	0	0.000000%
Chr09	68,611,519	68,611,019	500	0.000729%
Chr10	67,684,033	67,678,033	6,000	0.008865%
Chr11	66,824,821	66,821,821	3,000	0.004489%
Chr12	63,146,500	63,144,000	2,500	0.003959%
Total anchored	869,692,437 (96.80%)	869,647,937	44,500	0.005117%
Unanchored	28,726,195 (3.20%)

### Genome annotation and functional prediction

#### Identifying repeat sequences

The repeat sequences in the genome were identified using a combination of homologous sequence prediction and *ab initio* prediction. For homologous sequence prediction, RepeatMasker v1.323^37^ and RepeatProteinMask v1.36^38^ were used to predict the homology sequences against known repeat sequences in the database RepBase^39^. For *ab initio* prediction, RepeatModeler open-1.0.8^40^ was used to establish a *de novo* repeat sequence database, and RepeatMasker v1.323^37^ was used for prediction. Tandem Repeats Finder (TRF) v4.07b^41^ was used to find tandem repeat sequences in the genome. Combined with the results, 649.21 Mb repeat sequences were identified, accounting for 72.26% of the *S. rostratum* genome. The four predominant categories were long terminal repeats (LTR) (accounting for 46.06% of genome size), long interspersed nuclear elements (LINE) (3.62%), DNA elements (3.14%), and short interspersed nuclear elements (SINE) (0.22%) (Table 4).

**Table 4:** Statistics of repeat elements in the genome of *Solanum rostratum*.

Class	RepeatMasker TEs		RepeatProteinMask TEs		RepeatModeler TEs		Combined TEs	
Type	Length (bp)	% in genome	Length (bp)	% in genome	Length (bp)	% in genome	Length (bp)	% in genome
DNA	20,569,654	2.29	769,635	0.09	11,659,600	1.3	28,196,585	3.14
LINE	22,128,879	2.46	19,023,801	2.12	28,490,398	3.17	32,487,489	3.62
SINE	1,961,417	0.22	0	0	0	0	1,961,417	0.22
LTR	231,529,759	25.77	163,968,428	18.25	363,534,714	40.46	413,866,571	46.06
Unknown	34,056	0	0	0	234,827,738	26.14	234,857,657	26.14
Other	1,462,968	0.16	16,942,527	1.89	9,137,996	1.02	25,927,555	2.89
Total	277,121,683	30.84	200,396,923	22.3	624,136,628	69.47	649,214,848	72.26

Abbreviations: LINE, long interspersed nuclear element; SINE, short interspersed nuclear element; LTR, long terminal repeat.

#### Identifying non-coding RNA (ncRNA) gene

Rfam^42^ was used to predict ribosomal RNAs (rRNAs), small nuclear RNAs (snRNAs), and micro RNAs (miRNAs) by comparison with known non-coding RNA libraries. Transfer RNAs (tRNAs) were predicted using tRNAscan-SE v1.3.1^43^. In total, 3,588 ncRNAs were annotated in the *S. rostratum* genome, including 547 miRNAs, 1,288 tRNAs, 1,110 rRNAs, and 643 snRNAs (Table 5).

**Table 5:** Statistics for non-coding RNA genes in the genome of *Solanum rostratum*.

Class	Type	Numbers	Average length (bp)	Total length (bp)	Percentage in genome (%)
miRNA		547	96.73	52,910	0.00589
tRNA		1,288	75.25	96,925	0.01079
rRNA	18 S	84	603.13	50,663	0.00564
	28 S	82	132.06	10,829	0.00121
	5.8 S	30	134.53	4,036	0.00045
	5 S	914	119.64	109,349	0.01217
snRNA	CD-box	359	101.62	36,481	0.00406
	HACA-box	58	123.33	7,153	0.00080
	splicing	226	135.37	30,593	0.00341

#### Gene structure prediction

Three strategies were applied to predict the gene structure from the repeat-masked genome. The first strategy was homologous prediction. BLAST v2.2.28^44^ with an E-value cutoff of 1e-5 and GeMoMa v1.6^45^ were used to predict gene structure by comparing with seven closely related species (*C. annuum*^19^, *Solanum chilense*^46^, *Solanum commersonii*^47^, *S. lycopersicum*^16^, *S. melongena*^20^, *Solanum pennellii*^48^, and *S. tuberosum*^18^). The second strategy was based on transcriptome data. The filtered Illumina RNA-seq sequences from five libraries were assembled into transcripts using Trinity v2.11.0^49^ with default parameters. Then, the Trinity RNA-Seq assemblies and full-length cDNAs were aligned and mapped to the soft-masked genome assembly using GMAP v2014-10-2^50^ and BLAT Src35^51^. Candidate gene structures were extracted from the PASA v2.1^28^ pipeline based on the open reading frame (ORF). The third strategy included using *ab initio* prediction based on the characteristics of the genomic sequence data. Using Augustus v3.3^52^, SNAP v38926^53^, and GeneMark v4.33^54^, 29,485, 33,190, and 26,142 protein-coding genes were identified, respectively. Finally, EVM v1.11^27^ integrated the above three strategies, resulting in a non-redundant gene set, with weighting as default. Overall, 29,694 protein-coding genes were obtained, with an average gene length of 4,308 bp, cds length of 1,172 bp, exon length of 237 bp, and intron length of 795 bp (Table 6).

**Table 6:** Summary of gene structure prediction by three strategies of *Solanum rostratum*.

	Gene set	Number of proteins	Average gene length (bp)	Average cds length (bp)	Average exons per gene	Average exon length (bp)	Average intron length (bp)
*ab initio* prediction	Augustus	29,485	4,661.13	1,284.52	4.89	262.70	869.09
	GeneMark	33,190	4,931.98	1,127.19	5.35	210.83	876.39
	SNAP	26,142	17,230.72	596.34	3.93	151.56	5669.30
Transcriptome-based prediction	cDNA	27,408	7,687.74	1,493.68	6.93	387.13	845.39
Homology-based prediction	*Capsicum annuum*	32,842	3,902.16	1,137.54	4.76	239.12	736.83
	*Solanum chilense*	27,145	3,902.19	1,150.88	4.79	240.17	726.58
	*Solanum commersonii*	41,616	3,270.56	905.08	4.35	208.08	707.19
	*Solanum lycopersicum*	28,703	4,264.34	1,255.14	5.04	248.87	745.25
	*Solanum melongena*	32,600	3,497.00	1,018.97	4.47	227.85	714.71
	*Solanum pennellii*	29,777	4,275.29	1,252.46	5.03	249.07	751.35
	*Solanum tuberosum*	29,750	4,256.09	1,245.78	5.03	247.55	747.54
Total	EVM	29,694	4,308.30	1,171.80	4.95	236.83	795.49

#### Gene function annotation

Functional annotation of the protein-coding genes was carried out via BLAST^44^, with an E-value cutoff of 1e-5, against the public protein databases, including the Non-redundant protein database (NR) (ftp://ftp.ncbi.nlm.nih.gov/blast/db/FASTA/nr.gz), the nucleotide sequence database (NT) (https://www.ncbi.nlm.nih.gov/nucleotide/), SwissProt protein database (SwissProt)^55^, Kyoto Encyclopedia of Genes and Genomes (KEGG)^56^, Eukaryotic Orthologous Groups of proteins (KOG)^57^, and eggNOG-mapper v2.1.0-1^58^. Protein domains were predicted by searching against the Protein Families Database (Pfam)^59^ using Hmmer v3.1b1^60^ with default settings. Gene Ontology (GO)^61^ terms were obtained based on the corresponding InterPro^62^ or Pfam^59^ entry. A total of 28,154 genes (94.81%) were annotated using at least one public database (Table 7).

**Table 7:** Statistics for the *Solanum rostratum* functionally annotated protein-coding genes.

Database		Number	Percentage in genome (%)
Protein-coding genes		29,694	100
Annotated genes		28,154	94.81
	BLASTP	21,756	73.27
	BLASTX	21,542	72.55
	GO	21,987	74.05
	KEGG ID	8,047	27.10
	KEGG Pathway	5,062	17.05
	NR	27,830	93.72
	NT	26,451	89.08
	PFAM	21,703	73.09
	eggNOG	18,076	60.87
Unannotated genes		1,540	5.19

### Solanaceous orthology identification, phylogenetic tree construction, and divergence time estimation

Twenty solanaceous species were selected for comparative genomic analysis, with *Ipomoea trifida* as the outgroup. The longest transcripts, which were extracted using TBtools v1.106^63^, were used as the gene set for the following analysis. The orthogroups and orthologs classification were identified using Orthofinder v2.5.4^64^ with parameters -S diamond, -M msa, and -T fasttree. As a result, 824,030 genes (93.10% of total genes) were assigned to 56,426 orthogroups among 21 species, with 7,963 orthogroups shared in all the species and 799 shared single-copy orthogroups. Among the 29,694 genes in *S. rostratum*, 28,514 were clustered into 17,237 orthogroups, with 12,096 genes in single-copy orthologs, 16,418 genes in multiple-copy orthologs, and 1,065 genes in 298 species-specific orthogroups.

**Fig. 4:**
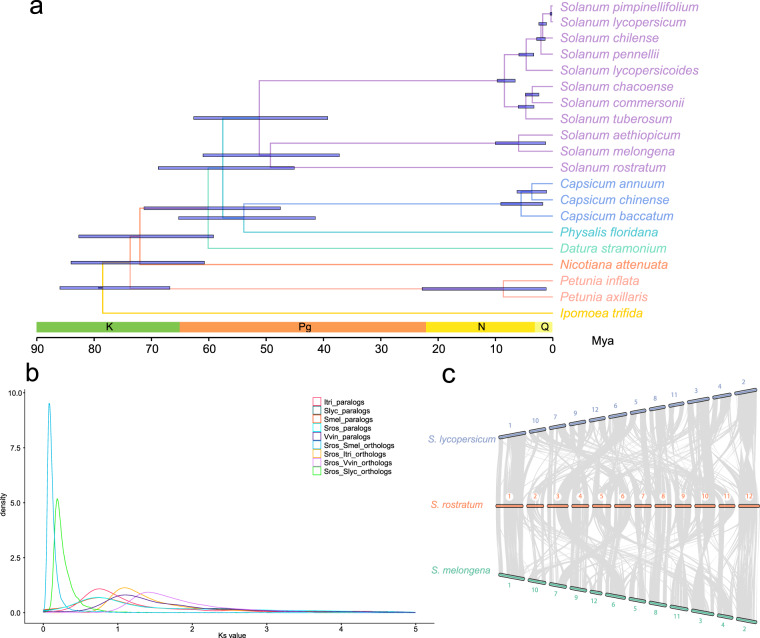
Comparative genomic and evolution analysis of solanaceous species. (**a**) Phylogenetic topology constructed based on shared single-copy genes, and divergence times estimation of solanaceous species with *Ipomoea trifida* as an outgroup. All the nodes supported bootstrap values are 100. The blue bars on the nodes represent the divergence time range with 95% confidence intervals (million years ago, Mya). The below scale represents the geologic time divisions, covering Cretaceous (K), Paleogene (Pg), Neogene (N), and Quaternary (Q). (**b**) Whole Genome Duplication events revealed by synonymous substitution rate (Ks) analysis. The Ks frequency density distributions of syntenic orthologous or paralogous block pairwise within and between genomes of *Solanum rostratum* (Sros) and *Vitis vinifera* (Vvin), *Ipomoea trifida* (Itri), *Solanum lycopersicum* (Slyc), and *Solanum melongena* (Smel). (**c**) Whole-genome synteny between *S. rostratum* and two other closely related *Solanum* species (*S. lycopersicum* and *S. melongena*). Conserved syntenic blocks are highlighted with grey color corresponding to the twelve pseudo-chromosomes, indicating visible genome rearrangements occurred during evolution among *Solanum* species.

A phylogenetic tree was constructed using the concatenated 799 single-copy orthogroup gene alignment generated using Orthofinder^64^. The maximum-likelihood method software raxmlHPC v8.2.12^65^ was implemented with the parameters -m PROTGAMMAJTT, -f a, and -# 100. The solanaceous tree recovered the monophyly of 3 subfamilies, 5 tribes, and 6 genera with 100 support values at all nodes, revealing a sister group relationship between *S. rostratum* and *S. melongena* + *S. aethiopicum* (Fig. 4a).

Four-fold Degenerate Synonymous Site (4DTv) was extracted from single-copy orthogroup genes to estimate the divergence time among Solanaceae using MCMCTree in PAML v4.10.3^66^ with the following parameters: clock = correlated rates, model = H85KY, alpha = 0.5, burn in = 100,000, sample frequency = 2, and sample number = 1,000,000. Two calibrations were set, which were obtained from Timetree^67^: the divergence time between Solanaceae and Convolvulaceae (59.1–83.9 million years ago [Mya]), and the divergence time between *S. lycopersicum* and *S. tuberosum* (6.1–9.0 Mya). The results revealed that *S. rostratum* split from the common ancestor ca. 49.26 Mya (Fig. 4a).

### WGD analysis

To investigate the WGD event history of Solanaceae, the synonymous substitution rate (Ks) frequency density distributions of syntenic orthologous block pairwise between genomes and syntenic paralogous block pairwise within genomes were calculated by wgd v1.1.2^68^, including *S. rostratum* (Sros), *Vitis vinifera*^69^ (Vvin), *Ipomoea trifida*^70^ (Itri), *S. lycopersicum*^16^ (Slyc), and *S. melongena*^20^ (Smel). For one-versus-one orthologs Ks distributions calculation, the module dmd was implemented to extract orthologs by all-versus-all blastp using the diamond^71^ algorithm with the parameters–nostrictcds -e 1e-10. The module ksd^66^ was then used to construct one-versus-one ortholog Ks distributions. For whole-paranome Ks distribution calculation, the module dmd was used to extract paralogs and cluster gene families using the Markov cluster (MCL)^72^ algorithm. Then, the module ksd^66^ was used to construct whole-paranome Ks distributions with the parameter -mp 1000. Finally, the module syn identified and extracted paralogs in intra-genomic colinear blocks using i-ADHoRe v3.0^73^. A shared peak was detected within Solanaceae at approximately 0.68, which occurred after the divergence peak with *V. vinifera*, and before the Solanaceae speciation peak, indicating that an an ancient WGD occurred in the ancestor of the Solanaceae. However, there was no subsequent WGD after species differentiation within the Solanaceae. Within Solanaceae, *S. rostratum* first diverged from *S. lycopersicum* at 0.14, and *S. melongena* at 0.03 (Fig. 4b).

### Whole-genome synteny

To understand the extend of genomic rearrangement of *S. rostratum* during evolution, whole-genome synteny analysis was conducted between *S. rostratum* (Sros) and *S. lycopersicum*^16^ (Slyc), and between *S. rostratum* and *S. melongena*^20^ (Smel). The protein sequences of Sros and Slyc, and Sros and Smel were blasted using blastp with parameter -evalue 1e-5. The multiple alignments of syntenic blocks were identified by MCScanX^74^ with the parameter -s 15 (number of genes required to call a collinear block) and visualized by jcvi v1.2.8^75^ with the parameter–minspan = 30. The complicated conserved syntenic blocks among the twelve pseudo-chromosomes, indicate that visible genome rearrangements occurred during evolution among *Solanum* (Fig. 4c).

## Data Records

All raw sequencing data have been deposited in the NCBI Sequence Read Archive (SRA) (Table 1) under Bioproject number PRJNA932047, including the genomic Illumina sequencing data (SRR23354532)^76^, genomic PacBio HiFi sequencing data (SRR23354533)^77^, transcriptome Illumina sequencing data (SRR23354526-SRR23354530)^78–82^, Hi-C sequencing data (SRR23354531)^83^, and transcriptome Pacbio-Sequel II sequencing data (SRR23354525)^84^.

The final chromosome-level assembled genome sequences were deposited in the NCBI Assembly database under Accession Number JARACL000000000^85^.

The genome annotation results, including repeated sequences, gene structure, and functional predictions were deposited in the Figshare database (10.6084/m9.figshare.22016024)^86^.

## Technical Validation

### Evaluation of the quality of genomic DNA and RNA

The purification, concentration, and integrity of the DNA template were quantitatively determined using a NanoDrop 8000 Spectrophotometer (Thermo Fisher Scientific, USA), Qubit Fluorometers (Thermo Fisher Scientific, USA), and Agilent 4200 Bioanalyzer (Agilent Technologies, USA), respectively. The evaluation results require the 15 kb insert library of the PacBio Sequel sequencing platform to meet the following criteria: including (1) the DNA content ≥ 10 μg, (2) the DNA concentration ≥ 80 ng/μL, (3) the DNA peak size was 32.59 kb which was over than 20 kb, (4) the DNA absorbance was 1.8 ≤ OD_260/280_ ≤ 2.0 and 1.6 ≤ OD_260/230_ ≤ 2.5.

The purification, concentration, and integrity of the RNA template were quantitatively determined using a NanoDrop 8000 Spectrophotometer (Thermo Fisher Scientific, USA), an Agilent 2100 Bioanalyzer (Agilent Technologies, USA), and an Agilent RNA 6000 Nano Kit (Agilent Technologies, USA), respectively. The evaluation results required all the meet for the Iso-seq library construction, including (1) the RNA content ≥ 4 μg, (2) the RNA concentration ≥ 250 ng/μL, (3) RNA Integrity Number (RIN) value ≥ 6.0, (4) the RNA absorbance was 2.0 ≤ OD_260/280_ ≤ 2.2, and 1.6 ≤ OD_260/230_ ≤ 2.1.

### Evaluating the completeness and quality of the genome assembly and annotation

#### Flow cytometry analysis

FACScalibur Flow cytometry (BD Biosciences, USA) analysis^87^ was conducted to estimate the *S. rostratum* genome size with three replicates, and ModFit software v5.0 (Yerity SoftwareHouse, USA) was used to analyze the results. The genome size of the internal reference standard *Glycine max* is 978.4 Mb (1 pg DNA = 0.978 G)^88^. The 2 C DNA content in pg of *S. rostratum* was calculated according to the following formula^89^: $S.rostratum\;2{\rm{C}}\;{\rm{DNA}}\;{\rm{content}}=\frac{{\rm{G}}1\;{\rm{peak}}\;{\rm{mean}}\;{\rm{of}}\;S.rostrarum\times C.max\;2{\rm{C}}\;{\rm{DNA}}\;{\rm{content}}}{{\rm{G}}1\;{\rm{peak}}\;{\rm{mean}}\;{\rm{of}}\;C.max}$. The peak values of *G. max* were 104.29, 106.54, and 103.70, respectively. The corresponding peak values for *S. rostratum* were 94.32, 97.10, and 94.28, respectively. The genome size of *S. rostratum* was estimated to be approximately 885.36–892.20 Mb, which was very close to the genome size of the pseudo-chromosome-level assembly in 898.42 Mb.

#### Mapped to the genome using Illumina data

Illumina paired-end reads were mapped back to the draft genome using Burrows-Wheeler Aligner (BWA) v0.7.9a^90^. Then, depth, mapping rates, and coverage at each position were calculated using samtools v0.1.19^91^. The results showed that 99.04% of read pairs were mapped to the genome with an average depth of 105.24 and a coverage rate of 97.54%, indicating high single-base concordance.

#### BUSCO assessment

**Fig. 5:**
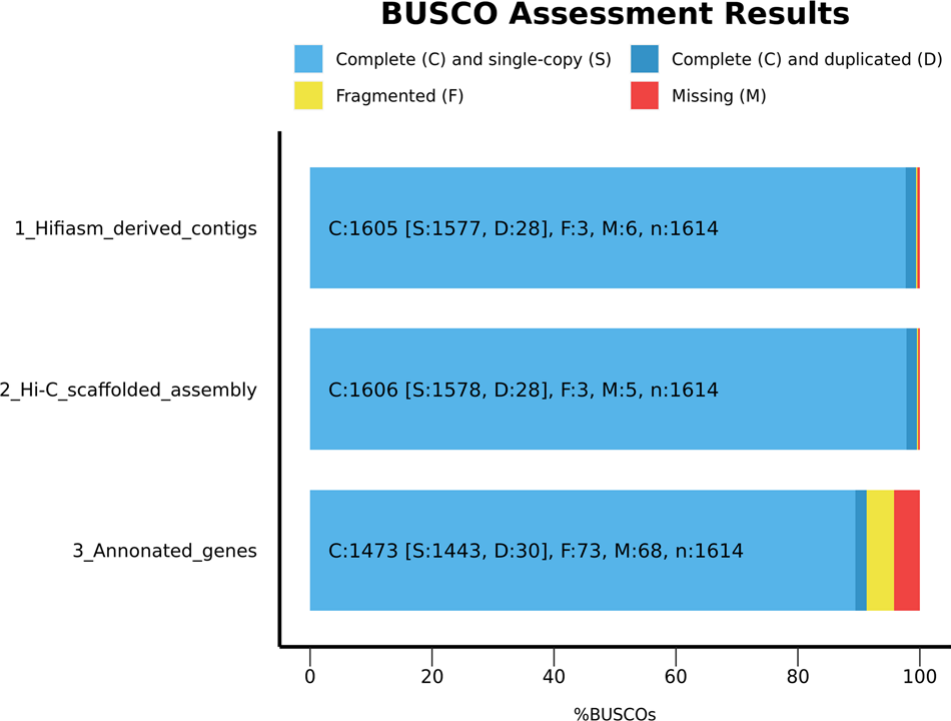
Benchmarking of genome completeness of *Solanum rostratum* genome assembly and annotation, evaluated by BUSCO based on embryophyta_odb10 database which includes 1,614 genes. C: the number of complete genes, S: the number of complete and single-copy genes, D: the number of complete and duplicated genes, F: the number of incomplete genes, M: the number of missing genes.

The completeness of the contig-level genome, Hi-C pseudo-chromosome-level genome, and predicted gene datasets were further evaluated with BUSCO (default parameters) v5.1.2^29^ based on the ortholog database embryophyta_odb10 (1,614 genes). The results were visualized by the python script generate_plot.py of BUSCO, showing a high completeness level with 99.4%, 99.5%, and 91.3% complete genes found in the contig-level genome, Hi-C pseudo-chromosome-level genome, and predicted gene datasets, respectively (Fig. 5).

#### Protein coding genes comparison with close species

**Fig. 6:**
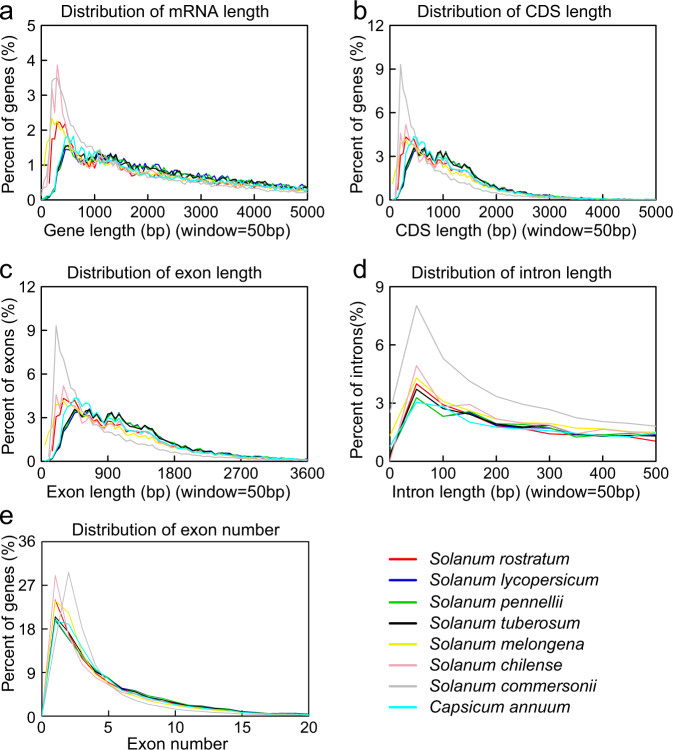
Annotated genes comparison of the distribution of (**a**) mRNA length (**b**) CDS length (**c**) exon length (**d**) intron length (**e**) exon number in *Solanum rostratum* with other closely related species. The x-axis represents the length or number and the y-axis represents the density of genes.

To determine the prediction accuracy and reliability, the distribution of mRNA length, CDS length, exon length, intron length, and exon number in *S. rostratum* and other closely related species (*C. annuum*^19^, *S. chilense*^46^, *S. commersonii*^47^, *S. lycopersicum*^16^, *S. melongena*^20^, *S. pennellii*^48^, and *S. tuberosum*^18^) were determined. The consistent distribution tendency among all species further supported an ideal annotated gene dataset in *S. rostratum* (Fig. 6).

Hence, a high-quality completeness and accuracy *S. rostratum* genome was assembled and annotated in the present study.

## Supplementary information

Supplementary Table 1

## Data Availability

No specific custom codes were developed in this study. All commands and pipelines used for data analyses were conducted according to the manuals or protocols provided by the corresponding software development team, which are described in detail in the Methods section. Default parameters were employed if no detailed parameters were mentioned for the software used in this study. Supplementary Table 1 lists the versions, settings, and parameters of the relevant software used in this study.

## References

[1] Randall, R. P. *A Global Compendium of Weeds*. (Department of Agriculture and Food Western Australia, 2012).

[2] Lin Y, Tan DY (2007). The potential and exotic invasive plant: *Solanum rostratum*. Acta Phytotaxonomiea Sinica.

[3] Bowers KA (1975). The pollination ecology of *Solanum rostratum* (Solanaceae). Am. J. Bot..

[4] Weber DC, Drummond FA, Ferro DN (1995). Recruitment of Colorado potato beetles (Coleoptera: Chrysomelidae) to solanaceous hosts in the field. Environ. Entomol..

[5] Mauricio-Castillo JA, Argüello-Astorga GR, Ambriz-Granados S, Alpuche-Solís AG, Monreal-Vargas CT (2007). First Report of Tomato golden mottle virus on *Lycopersicon esculentum* and *Solanum rostratum* in Mexico. Plant Dis..

[6] Singh RP, Bagnall RH (1968). *Solanum rostratum* Dunal., a new test plant for the potato spindle tuber virus. Am. J. Potato Res..

[7] Bassett IJ, Munro DB (1986). The biology of Canadian weeds.: 78. *Solanum carolinense* L. and *Solanum rostratum* Dunal. Can. J. Plant Sci..

[8] Wei S (2009). Factors affecting buffalobur (*Solanum rostratum*) seed germination and seedling emergence. Weed Sci..

[9] USDA-NRCS. The PLANTS Database. *National Plant Data Center* http://plants.usda.gov/ (2014).

[10] (2022). GBIF Secretariat.

[11] Guan G (1984). *Solanum rostratum* - A quarantine weed. Plant Quarantine.

[12] Kane NC, Rieseberg LH (2008). Genetics and evolution of weedy *Helianthus annuus* populations: adaptation of an agricultural weed. Mol. Ecol..

[13] Marks RA, Hotaling S, Frandsen PB, VanBuren R (2021). Representation and participation across 20 years of plant genome sequencing. Nat. Plants.

[14] Sun Y, Shang L, Zhu QH, Fan L, Guo L (2021). Twenty years of plant genome sequencing: achievements and challenges. Trends Plant Sci..

[15] PBI Solanum Project. Solanaceae Source website. *USA: Planetary Biodiversity Inventories (PBI), National Science Foundation* http://www.solanaceaesource.org/ (2014).

[16] Hosmani, P.S. *et al*. An improved *de novo* assembly and annotation of the tomato reference genome using single-molecule sequencing, Hi-C proximity ligation and optical maps. *BioRxiv*, 767764 (2019).

[17] Takei H (2021). *De novo* genome assembly of two tomato ancestors, *Solanum pimpinellifolium* and *Solanum lycopersicum* var. *cerasiforme*, by long-read sequencing. DNA Res..

[18] Pham GM (2020). Construction of a chromosome-scale long-read reference genome assembly for potato. GigaScience.

[19] Qin C (2014). Whole-genome sequencing of cultivated and wild peppers provides insights into *Capsicum* domestication and specialization. Proc. Natl. Acad. Sci. USA.

[20] Barchi L (2021). Improved genome assembly and pan-genome provide key insights into eggplant domestication and breeding. Plant J..

[21] Sierro N (2014). The tobacco genome sequence and its comparison with those of tomato and potato. Nat. Commun..

[22] Lu J (2021). The *Physalis floridana* genome provides insights into the biochemical and morphological evolution of *Physalis* fruits. Hortic. Res..

[23] Rajewski A, Carter-House D, Stajich J, Litt A (2021). *Datura* genome reveals duplications of psychoactive alkaloid biosynthetic genes and high mutation rate following tissue culture. BMC Genomics.

[24] Cao YL (2021). Wolfberry genomes and the evolution of *Lycium* (Solanaceae). Commun. Biol..

[25] Cheng H, Concepcion GT, Feng X, Zhang H, Li H (2021). Haplotype-resolved *de novo* assembly using phased assembly graphs with hifiasm. Nat Methods.

[26] Servant N (2015). HiC-Pro: An optimized and flexible pipeline for Hi-C processing. Genome Biol..

[27] Haas BJ (2008). Automated eukaryotic gene structure annotation using EVidenceModeler and the Program to Assemble Spliced Alignments. Genome Biol..

[28] Haas BJ (2003). Improving the *Arabidopsis* genome annotation using maximal transcript alignment assemblies. Nucleic Acids Res..

[29] Seppey, M., Manni, M. & Zdobnov, E.M. BUSCO: assessing genome assembly and annotation completeness. In Gene prediction, M. Kollmar, ed. (New York, USA: Springer), pp. 227-245 (2019). 10.1007/978-1-4939-9173-0_14 31020564

[30] Allen GC, Flores-Vergara MA, Krasynanski S, Kumar S, Thompson WF (2006). A modified protocol for rapid DNA isolation from plant tissues using cetyltrimethylammonium bromide. Nat. Protoc..

[31] Chen S, Zhou Y, Chen Y, Gu J (2018). fastp: an ultra-fast all-in-one FASTQ preprocessor. Bioinformatics.

[32] Rio DC, Ares M, Hannon GJ, Nilsen TW (2010). Purifcation of RNA using TRIzol (TRI reagent). Cold Spring Harbor Protocols.

[33] Wood DE, Lu J, Langmead B (2019). Improved metagenomic analysis with Kraken 2. Genome Biol..

[34] Langmead B, Salzberg S (2012). Fast gapped-read alignment with Bowtie 2. Nat Methods.

[35] Rice A (2015). The Chromosome Counts Database (CCDB) - a community resource of plant chromosome numbers. New Phytol..

[36] Akdemir KC, Chin L (2015). HiCPlotter integrates genomic data with interaction matrices. Genome Biol..

[37] Tempel S (2012). Using and Understanding RepeatMasker. Methods Mol. Biol..

[38] Tarailo‐Graovac M, Chen N (2009). Using RepeatMasker to identify repetitive elements in genomic sequences. Curr. Protoc. Bioinform..

[39] Bao W, Kojima KK, Kohany O (2015). Repbase Update, a database of repetitive elements in eukaryotic genomes. Mob DNA.

[40] Flynn JM (2020). RepeatModeler2 for automated genomic discovery of transposable element families. Proc. Natl. Acad. Sci. USA.

[41] Benson G (1999). Tandem repeats finder: a program to analyze DNA sequences. Nucleic Acids Res..

[42] Griffiths-Jones S (2003). Rfam: an RNA family database. Nucleic Acids Res..

[43] Lowe TM, Eddy SR (1997). tRNAscan-SE: a program for improved detection of transfer RNA genes in genomic sequence. Nucleic Acids Res..

[44] McGinnis S, Madden TL (2004). BLAST: at the core of a powerful and diverse set of sequence analysis tools. Nucleic Acids Res..

[45] Keilwagen, J., Hartung, F. & Grau, J. GeMoMa: Homology-based gene prediction utilizing intron position conservation and RNA-seq data. In Gene prediction, Kollmar, M. ed. (New York, USA: Springer), pp. 161-177 (2019). 10.1007/978-1-4939-9173-0_9 31020559

[46] Stam R (2019). The *de novo* reference genome and transcriptome assemblies of the wild tomato species *Solanum chilense* highlights birth and death of NLR genes between tomato species. G3.

[47] (2023). National Center for Biotechnology Information.

[48] Bolger A (2014). The genome of the stress-tolerant wild tomato species *Solanum pennellii*. Nat. Genet..

[49] Haas BJ (2013). *De novo* transcript sequence reconstruction from RNA-seq using the Trinity platform for reference generation and analysis. Nat Protoc..

[50] Wu TD, Watanabe CK (2005). GMAP: a genomic mapping and alignment program for mRNA and EST sequences. Bioinformatics.

[51] Kent WJ (2002). BLAT–the BLAST-like alignment tool. Genome Res..

[52] Stanke M, Steinkamp R, Waack S, Morgenstern B (2004). AUGUSTUS: a web server for gene finding in eukaryotes. Nucleic Acids Res..

[53] Leskovec J, Sosič R (2016). Snap: A general-purpose network analysis and graph-mining library. ACM T. Intel. Syst. Tec..

[54] Ter-Hovhannisyan V, Lomsadze A, Chernoff YO, Borodovsky M (2008). Gene prediction in novel fungal genomes using an *ab initio* algorithm with unsupervised training. Genome Res..

[55] Boeckmann B (2003). The SWISS-PROT protein knowledgebase and its supplement TrEMBL in 2003. Nucleic Acids Res..

[56] Kanehisa M, Furumichi M, Sato Y, Ishiguro-Watanabe M, Tanabe M (2021). KEGG: integrating viruses and cellular organisms. Nucleic Acids Res..

[57] Tatusov RL (2003). The COG database: an updated version includes eukaryotes. BMC Bioinformatics.

[58] Huerta-Cepas J (2019). eggNOG 5.0: a hierarchical, functionally and phylogenetically annotated orthology resource based on 5090 organisms and 2502 viruses. Nucleic Acids Res..

[59] Finn, R.D. *et al*. Pfam: the protein families database. *Nucleic Acids Res*. 42(Database issue), 222-30 (2014). 10.1093/nar/gkt1223 PMC3965110 24288371

[60] Eddy SR (2011). Accelerated profile HMM searches. PLoS Comput. Biol..

[61] Ashburner M (2000). Gene ontology: tool for the unification of biology. Nat. Genet..

[62] Paysan-Lafosse T (2023). InterPro in 2022. Nucleic Acids Res..

[63] Chen C (2020). TBtools: an integrative toolkit developed for interactive analyses of big biological data. Mol. Plant.

[64] Emms DM, Kelly S (2019). OrthoFinder: phylogenetic orthology inference for comparative genomics. Genome Biol..

[65] Stamatakis A (2014). RAxML version 8: a tool for phylogenetic analysis and post-analysis of large phylogenies. Bioinformatics.

[66] Yang ZH (2007). PAML 4: Phylogenetic Analysis by Maximum Likelihood. Mol. Biol. Evol..

[67] Benton MJ, Donoghue PCJ, Asher RJ (2009). Calibrating and constraining molecular clocks. The Timetree of Life.

[68] Zwaenepoel A, Van de Peer Y (2019). wgd - simple command line tools for the analysis of ancient whole genome duplications. Bioinformatics.

[69] The French-Italian Public Consortium for Grapevine Genome Characterization (2007). The grapevine genome sequence suggests ancestral hexaploidization in major angiosperm phyla. Nature.

[70] Hirakawa H (2015). Survey of genome sequences in a wild sweet potato, *Ipomoea trifida* (HBK) G. Don. DNA Res..

[71] Buchfink B, Xie C, Huson DH (2014). Fast and sensitive protein alignment using diamond. Nat Methods.

[72] van Dongen S.M. Graph Clustering by Flow Simulation. PhD Thesis, University of Utrecht, Utrecht, The Netherlands (2000).

[73] Proost S (2012). i-ADHoRe 3.0: fast and sensitive detection of genomic homology in extremely large data sets. Nucleic Acids Res..

[74] Wang YP (2012). MCScanX: a toolkit for detection and evolutionary analysis of gene synteny and collinearity. Nucleic Acids Res..

[75] Tang H (2008). Synteny and collinearity in plant genomes. Science.

[76] (2023). NCBI Sequence Read Archive.

[77] (2023). NCBI Sequence Read Archive.

[78] (2023). NCBI Sequence Read Archive.

[79] (2023). NCBI Sequence Read Archive.

[80] (2023). NCBI Sequence Read Archive.

[81] (2023). NCBI Sequence Read Archive.

[82] (2023). NCBI Sequence Read Archive.

[83] (2023). NCBI Sequence Read Archive.

[84] (2023). NCBI Sequence Read Archive.

[85] Zhang Y (2023). GenBank.

[86] Zhang Y (2023). figshare.

[87] Duda M, Gasińska A, Gregoraszczuk E (1999). Flow cytometric cell cycle analysis of two subpopulations of porcine granulosa cells. Exp. Clin. Endocrinol. Diabetes..

[88] Valliyodan B (2019). Construction and comparison of three reference-quality genome assemblies for soybean. Plant J..

[89] Doležel J, Bartoš JAN (2005). Plant DNA flow cytometry and estimation of nuclear genome size. Ann. Bot-London.

[90] Li, H. Aligning sequence reads, clone sequences and assembly contigs with BWA-MEM. *arXiv e-prints* (2013).

[91] Danecek P (2021). Twelve years of SAMtools and BCFtools. Gigascience.

