# Supplementary material for: Chromosome-level genome assembly and annotation of the prickly nightshade Solanum rostratum Dunal
Source: Sci Data. 2023 Jun 1;10:341. doi: 10.1038/s41597-023-02247-3 (PMC10235051; doi:10.1038/s41597-023-02247-3)
Supplement: Supplementary file 1 — Supplementary Table 1 [file 41597_2023_2247_MOESM1_ESM.docx]

Supplementary Table 1. Software used in the present study

| Software | Usage | Version | Parameters/Commands |
| --- | --- | --- | --- |
| fastp | data quality control and filter | 0.12.4 | default |
| ccs | extract HiFi reads from subreads | 6.0.0 | --maxLength = 50000 --minPasses = 3 --minPredictedAccuracy = 0.99 |
| lima | demultiplex and remove barcodes and primers | 2.1.0 | default |
| iso-seq3 | remove polyA tail and artificial concatemers; cluster and polish | 3.4.0 | default |
| bam2fastx | convert BAM file to gzipped fastq file | 1.3.1 | default |
| Hifiasm | *de novo* assembly for HiFi reads | 0.16.0 | default parameter |
| Kraken2 | check for potential contaminant sequences | 2.1.2 | default parameter |
| Bowtie2 | align Hi-C sequencing data on the contig assembly | 2.2.5 | --very-sensitive -L 20 --score-min L,-0.6,-0.2 --end-to-end --reorder --rg-id BMG --phred33-quals -p 5 |
| HiC-Pro | detect valid ligation products; generate intra- and inter- chromosomal contact maps | 2.7.8 | LIGATION_SITE = GATCGATC |
| HiCPlotter | HiC interaction matrice visualization | 0.6.6 | -r 50000 -tri 1 |
| RepeatMasker | screen repeat sequences against RepBase | 1.323 | -e ncbi |
| RepeatProteinMask | screen repeat sequences against RepBase | 1.36 | default |
| RepeatModeler | *de novo* transposable element family identification | open-1.0.8 | -engine ncbi |
| TRF | detect tandem repeats | 4.07b | 2 7 7 80 10 50 500 -f -d -h -r |
| tRNAscan-SE | predict tRNA genes | 1.3.1 | default |
| blast | sequence similarity search by alignment | 2.2.28 | evalue = 1e-5 |
| GeMoMa | homology-based gene prediction | 1.6 | default |
| Trinity | Illumina RNA-Seq *de novo* assembly | 2.11.0 | default |
| GMAP | map and align cDNA sequences to genome | 2014-10-2 | default |
| BLAT | map and align cDNA sequences to genome | Src35 | default |
| PASA | transcriptome-based gene prediction | 2.1 | -m = 50 |
| Augustus | initio-based gene prediction | 3.3 | --genemodel = partial |
| SNAP | initio-based gene prediction | 38926 | default |
| GeneMark | initio-based gene prediction | 4.33 | default |
| EVM | combine all the predictions into weighted consensus gene structures | 1.11 | default |
| HMMER | search against Pfam to predict protein domains | 3.1b1 | default |
| [eggNOG-mapper](https://github.com/eggnogdb/eggnog-mapper/wiki/eggNOG-mapper-v2.1.2-to-v2.1.4) | functional annotation, orthology assignment, and domain prediction | 2.1.0-1 | --itype proteins -m diamond |
| TBtools | extract longest transcripts; draw the Circos of genomic landscape | 1.106 | default |
| Orthofinder | orthogroups and orthologs classification | 2.5.4 | -S diamond -M msa -T fasttree |
| raxmlHPC | Phylogenetic inference with the maximum likelihood method | 8.2.12 | -m PROTGAMMAJTT -f a -# 100 |
| PAML | estimate the divergence time by MCMCTree | 4.10.3 | clock = correlated rates, model = H85KY, alpha = 0.5, burn in = 100,000, sample frequency = 2, sample number = 1,000,000 |
| CAFE | analyze changes in gene family size | 4.2 | -p 0.01 -s -t |
| MCScanX | detect syntenic blocks | - | -s 15 |
| jcvi | visualize syntenic blocks | 1.2.8 | --minspan=30 |
| diamond | all-versus-all blastp | - | --nostrictcds -e 1e-10 |
| MCL | cluster sequences in paralogous gene families | - | default |
| i-ADHoRe | identify and extract paralogs in intragenomic colinear blocks | 3.0 | default |
| BWA | mapp Illumina reads against genome | 0.7.9a | mem -M -k 30 |
| samtools | calculate depth, mapping rates, and coverage | 0.1.19 | default parameter |
| BUSCO | evaluate the assembly and annotation completeness and quality | 5.1.2 | -l embryophyta_odb10 |
